# Modeling cometabolism of hexavalent chromium by iron reducing bacteria in tertiary substrate system

**DOI:** 10.1038/s41598-021-90137-2

**Published:** 2021-05-25

**Authors:** Shivangi Upadhyay, Alok Sinha

**Affiliations:** Department of Environmental Science & Engineering, Indian Institute of Technology (ISM) Dhanbad, Dhanbad, Jharkhand 826 004 India

**Keywords:** Biotechnology, Microbiology, Environmental sciences, Nanoscience and technology

## Abstract

In this study, a bacterial strain *Serratia* sp. was employed for the reduction of synthetically prepared different concentration of Cr(VI) solution (10, 25, 40, 50 and 100 mg/L). Cometabolism study have been carried out in the binary substrate system as well as in the tertiary substrate system. The results revealed that when glucose was added as a co-substrate, at low Cr(VI) concentration, complete reduction was achieved followed by increased biomass growth, but when Cr(VI) concentration was increased to 100 mg/L, the reduction decline to 93%. But in presence of high carbon iron filings (HCIF) as co-substrate even at higher Cr(VI) concentration i.e. 100 mg/L, 100% reduction was achieved and the cell growth continued till 124 h. The study was illustrated via Monod growth kinetic model for tertiary substrate system and the kinetic parameters revealed that the HCIF and glucose combination showed least inhibition to hexavalent chromium reduction by *Serratia* sp.

## Introduction

A prevalent environmental pollutant chromium, is widely used in different industries such as cement, tannery, smelting industries and in electroplating^1^ for various purposes^2,3^. The presence of chromium mostly occurs in two different oxidation form i.e. hexavalent and trivalent one that signifies their toxicity. Amongst the two, the former one is highly toxic because of its higher solubility^4^ penetrable capability through biological membranes, highly mobile and have carcinogenic, mutagenic property^5^ and also intramolecular nucleic acid interaction, whereas, the latter one is less toxic, act as an important element in trace amount^5^ and important in metabolism of carbohydrate and lipids^6^. Having mutagenic and carcinogenic effects on humans such as internal hemorrhage, nausea, dermatitis, asthma, liver and kidney damage^7^ based on this, the powerful pollutant has been recognized as type I carcinogen and been further assigned the limit of total chromium presence in drinking water to less than 0.1 mg L^−1^ by the International Agency for Research on Cancer (IARC) and the United States Environmental Protection Agency (USEPA) respectively^8^. Cr(VI) reduction through conventional methods like chemical reduction, reverse osmosis, ion exchange, precipitation, adsorption and coagulation have major drawbacks including generation of secondary pollutants and high operating cost^9–11^. High carbon iron filings (HCIF) may offer the best choice as a reactive media due to its non-toxicity, low cost and availability. HCIF is capable of adsorbing, reducing and transforming many chlorinated hydrocarbons, heavy metals, and nitroaromatic compounds. A study by^10^ demonstrated the use of HCIF in Cr(VI) reduction and the results revealed that Cr(VI) adsorption to graphite inclusions that are present on HCIF had a great role in reduction of aqueous concentration. An experiment conducted by^12^ for effective removal of Cr(VI) from wastewater using ultrasonic pretreated sludge derived stable magnetic active carbon. The synthesized UMC had a high proportion of ZVI. Both the ZVI and carbon in the UMC was disclosed to be the domain electron donors for treating Cr(VI)-containing wastewater having concentration 2 mg/L. Another method of Cr(VI) removal was adopted by^13^ using Fluorine and nitrogen co-doped magnetic carbons (FN-MCs) which showed good removal efficiency. But the above mentioned methods have certain shortcomings like generation of secondary pollutants and high operational cost. An eco-friendly approach towards treatment of hexavalent chromium is biological reduction just before the disposal of wastewater into the environment by industries^14^. There are a number of microorganisms involved in the process of reduction and has been proved to be effective in this context^15–18^. But some of the forces hinders the overall process of microbial reduction. Focus should be given on reducing the hindrance such as adequate amount of nitrogen and carbon source, suitable pH and temperature suitability of wastewater, and the other toxic heavy metals presence in wastewater. These problems create difficulty for the non-indigenous microorganisms to effectively treat the wastewater.

Cometabolic degradation has been found to be eco-friendly and economically feasible in the process of treating recalcitrant compounds^19^, and coking wastewater (CWW) treatment^20^. Many studies stated that when phenol was used as the cometabolic substrate, the degradation efficiency of *p*-nitrophenol^21^, carbazole^22^ and 4-chlorophenol^23^ increased. In cometabolism, metabolic substrates such as glucose, methyl alcohol are sufficient source of carbon and energy for microbes to grow. These substrates induces enzymes synthesis that accelerates metabolism of growth and non-growth substrates, producing NADH as electron donor in metabolic pathway and subsequently these metabolites take part in degradation of various compounds^24^. Little attention has been given to cometabolic reduction of hexavalent chromium.

Earlier in our previous work, *Serratia s*p. was able to reduce different range of hexavalent chromium. But the inhibition effect of hexavalent chromium was higher for *Serratia* sp. As a result, the growth of the bacterial species was hindered after few hr^25^. To reduce the inhibitory effect of Cr(VI) on the bacteria, this study demonstrates the use of glucose and high carbon iron filings (HCIF) as cometabolic substrates for microbial reduction of Cr(VI).

## Materials and methods

### Chemicals

The chemicals, reagents and microbial media were of pure grade. Cr(VI) standard solution of 1000 mg L^−1^ have been prepared by mixing K_2_CrO_7_ in Milli Q water. For measuring hexavalent chromium concentration, Diphenyl carbazide (DPC) solution (0.25%) was prepared by dissolving it in 95%. Microbes were grown in broth media of Lureia Bertani (Hi-Media India). Glucose (Rankem, purity 99.0%) were used as cometabolic substrates.

### Experimental process

Bacterial strain was isolated from iron ore mines as discussed in the work done previously by^25^. Commercially available cast iron rod was chipped on a lathe machine and was brought into iron filings in a dough-sized ball mill. HCIF thus obtained were washed with N_2_ sparged 1 N HCl for 4–5 times so that the organic material adsorbed on HCIF surface during chipping process may be removed as well as the reactivity of HCIF may be enhanced. Afterwards, HCIF was washed again with N_2_ sparged milli Q water for 10–12 times to remove excess HCl. Later, moisture content of HCIF was removed by washing with 95% acetone and HCIF were dried in N_2_ atmosphere in a vacuum desiccator and then used for the experimental purpose. Cometabolism studies were conducted for enhancing the reduction capacity of the HCIF-bacteria system. Batch experiments were conducted with two different combination (i) Binary system containing bacteria and glucose (1 g/250 mL) and (ii) Tertiary system with bacteria, HCIF (1 g/250 mL) and glucose (1 g/250 mL) having different Cr(VI) concentration (10, 25, 40, 50, and 100 mg/L) in an incubator shaker at 35 °C and 120 rpm. Samples were taken at every 12 h and analysis of Cr(VI), biomass, and glucose concentration was measured by Uv–Vis spectrophotometer at 540, 600 and 510 nm, respectively. Samples were taken at regular time and concentration of Cr(VI), bacterial growth, and glucose was measured by Uv–Vis spectrophotometer at wavelength of 540, 600 and 510 nm respectively.

### Rate kinetics model

The pseudo first and second order was applied to define the kinetic degradation of microbe’s kinetics of hexavalent chromium which is given below:

1 $$C={C}_{O{e}^{-kt}}$$

where, C is the concentration at any time, initial concentration is denoted as C_O_, k is the pseudo first-order rate constant (day^−1^), and reaction time is denoted by t.

### Kinetics of microbial growth in system containing single substrate

Growth kinetics in single system comprises of bacteria and glucose and chromium exposure. In a batch system, the specific growth rate of a cell, µ (h^−1^) is defined as

2 $$\upmu = \frac{1}{X}\frac{dX}{dt}=\frac{dlnX}{dt}$$

where, µ is calculated at the exponential phase of the growth curve. X is the concentration of cell in either cell number at time ‘t’ based on the viable counts (CFU/mL) or in either g/L (dry basis). The specific growth rate of cell on single substrate is expressed in terms of µ in Eq. (2), which is a function of concentration of resource. The Haldane Andrew model was applied here in the single substrate system as it has its wide use in representing the growth kinetics of single substrate:

3 $$\upmu =\upmu maxS/ \left(S+\frac{{S}^{2}}{{K}_{I}}+ {K}_{S}\right)$$

where, µ_max_ is the maximum specific growth rate (day^−1^), substrate concentration is defined by the term ‘S’ and K_s is_ the substrate affinity constant (mg/L).

### Growth kinetics in tertiary substrate system

To stimulate Co-metabolism, for the cell growth on mixtures of substrate, various models have been proposed. In this study, Monod model used in binary substrate system^19^ was modified to tertiary substrate system for the analysis of growth kinetic parameters. The equation for the specific growth rate on tertiary inhibitory substrates was as follows:

4 $$\upmu =\frac{{\upmu }_{maxC}{S}_{C}}{{K}_{SC}+ {S}_{C}+{K}_{2 C }{S}_{P}+{K}_{3 C}{S}_{h}+{K}_{4C}{S}_{P}{S}_{h}}+\frac{{\upmu }_{maxP}{S}_{P} }{{K}_{SP}+{S}_{C}+{K}_{2P}{S}_{C}+{K}_{3P}{S}_{h}+{K}_{4P}{S}_{C}{S}_{h}}+\frac{{\upmu }_{maxh}{S}_{h}}{{K}_{Sh}+{S}_{h}+{K}_{2h}{S}_{C}+{K}_{3h}{S}_{P}+{K}_{4h}{S}_{C}{S}_{P}}$$

where the subscripts C, P, and h represents glucose as a carbon source, Cr(VI) and HCIF respectively. The physical meaning of *K*_*s*_ and µ_max_ is basically the same as described in Eq. (4) and obtained in single system. µ_maxC_S_C,_ denotes maximum specific growth rate in presence of glucose which was obtained from the results of growth kinetics in binary substrate system, Similarly, µ_maxP_S_P, is_ denoted by maximum specific growth rate in presence of chromium and µ_maxh_S_h,_ is denoted by maximum specific growth rate in presence of HCIF which were obtained from the model fitting of growth kinetics in binary substrate system. Equation (4) implies that there are kinetic interactions between all the three substrates if all K_2i,_ K_3i_, and K_4i_ (i = C, P and h) are not equal to zero. Here, the literal meaning of K_2_, K_3_ and K_4_ is described below:

- ${K}_{2 C }{S}_{P}$ = Inhibition of glucose in the presence of Cr(VI)
- ${K}_{3 C}{S}_{h}$ = Inhibition of glucose in the presence of HCIF
- ${K}_{4C}{S}_{P}{S}_{h}$ = Inhibition of glucose in the presence of Cr(VI) and HCIF
- ${K}_{2P}{S}_{C}$ = Inhibition of Cr(VI) in the presence of glucose
- ${K}_{3P}{S}_{h}$ = Inhibition of Cr(VI) in the presence of HCIF
- ${K}_{4P}{S}_{C}{S}_{h}$ = Inhibition of Cr(VI) in the presence of glucose and HCIF
- ${K}_{2h}{S}_{C}$ = Inhibition of HCIF in the presence of glucose
- ${K}_{3h}{S}_{P}$ = Inhibition of HCIF in the presence of Cr(VI)
- ${K}_{4h}{S}_{C}{S}_{P}$ = Inhibition of HCIF in the presence of glucose and Cr(VI).

### Statistical analysis

The experiments have been performed in triplicates and mean of three samples were taken. To determine the mean and standard deviation of the data sets, XLSTAT package of Microsoft excel 2013 was used. One way ANOVA followed by Duncan’s post hoc test was performed to determine the biomass concentration of the test isolate in absence and presence of chromium.

## Results and discussion

### Reduction of Cr(VI) in batch reactors in the presence of cometabolic substrate (HCIF and glucose)

**Figure 1:**
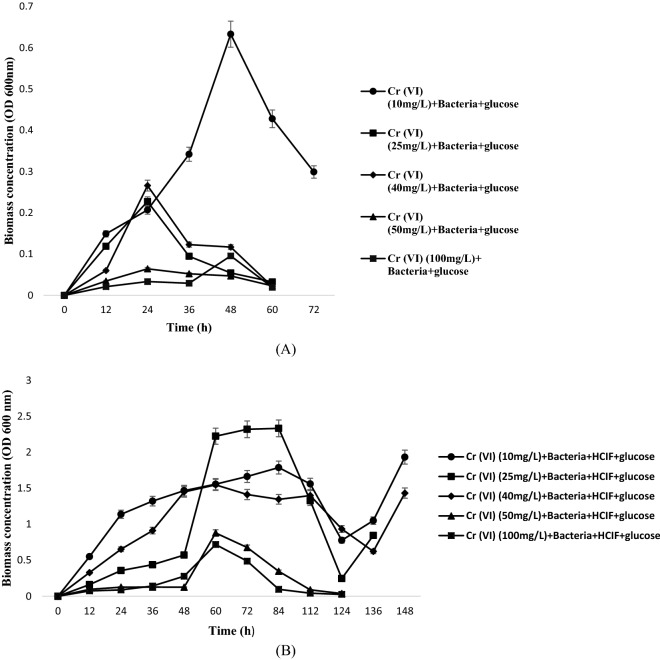
Temporal variation in biomass concentration with the (**A**) binary substrate system (**B**) tertiary substrate system.

The strain isolated was identified as *Serratia* sp.^25^. It was used in our previous study for reduction of different concentration of Cr(VI) in two different ways, i.e. one by the strain alone and the other by co-assistance of HCIF with the strain. This study was performed to demonstrate the cometabolic effect of substrate on reduction of hexavalent chromium. Cometabolism study was conducted to evaluate the cometabolic activity of *Serratia* sp. in two different sets i.e. when glucose was added (1 g/250 mL) to batch reactors containing Cr(VI) and bacteria (Set 1) and when glucose was added to batch reactors containing Cr(VI), bacteria and HCIF (Set 2) for the reduction of hexavalent chromium by Serratia sp. The biomass concentration, Cr(VI) concentration and glucose concentration was measured in the two sets. Figure 1A,B, shows the biomass abundance at different concentration of Cr(VI) (10, 25, 40, 50, and 100 mg/L) when glucose was added as cometabolic substrate in both the sets. The results of Set 1 indicated that when glucose was added to batch reactors containing only bacteria and Cr(VI), the bacterial growth was highest for 10 mg/L Cr(VI) solution, the growth increased till 50 h and after which it started declining. The lesser the concentration of Cr(VI), the higher was the bacterial growth. For 25 mg/L Cr(VI) concentration, the growth was highest at 24 h and then it declined and lasted till 60 h. In case of 50 mg/L of Cr(VI) concentration, the biomass growth was very low in comparison to other concentrations. However, slight increase in growth was observed at 24 h. In case of 100 mg/L of Cr(VI) concentration, the bacteria attained its log phase at 50 h and after that the stationary phase was observed. In this case the lag phase increased with increase in initial Cr(VI) concentration which may be due to the inhibitory effect of Cr(VI) on the growth of microorganism. At high concentrations, the inhibitory effect increased due to the fact that a fixed amount of inoculum was used for all different concentration of Cr(VI). In the second experimental set (Set 2), it was found out that at all the different concentration of Cr(VI) (10, 25, 40, 50 and 100 mg/L) the log phase started after 50 h and the cell doubling continues till 85 h. After this the decline in biomass concentration was observed. The results from the study indicated that when only glucose acted as co-metabolic substrate, the biomass growth lasted till 60 h but when glucose was added to the HCIF and bacterial set, the bacterial growth continues till 120 h. This may be due to the reason that, HCIF may be acting as a co-metabolic substrate in enhancing the bacterial growth along with the glucose. HCIF too acts as a source of growth to bacteria like glucose, when supplied with low dosing^26^.

**Figure 2:**
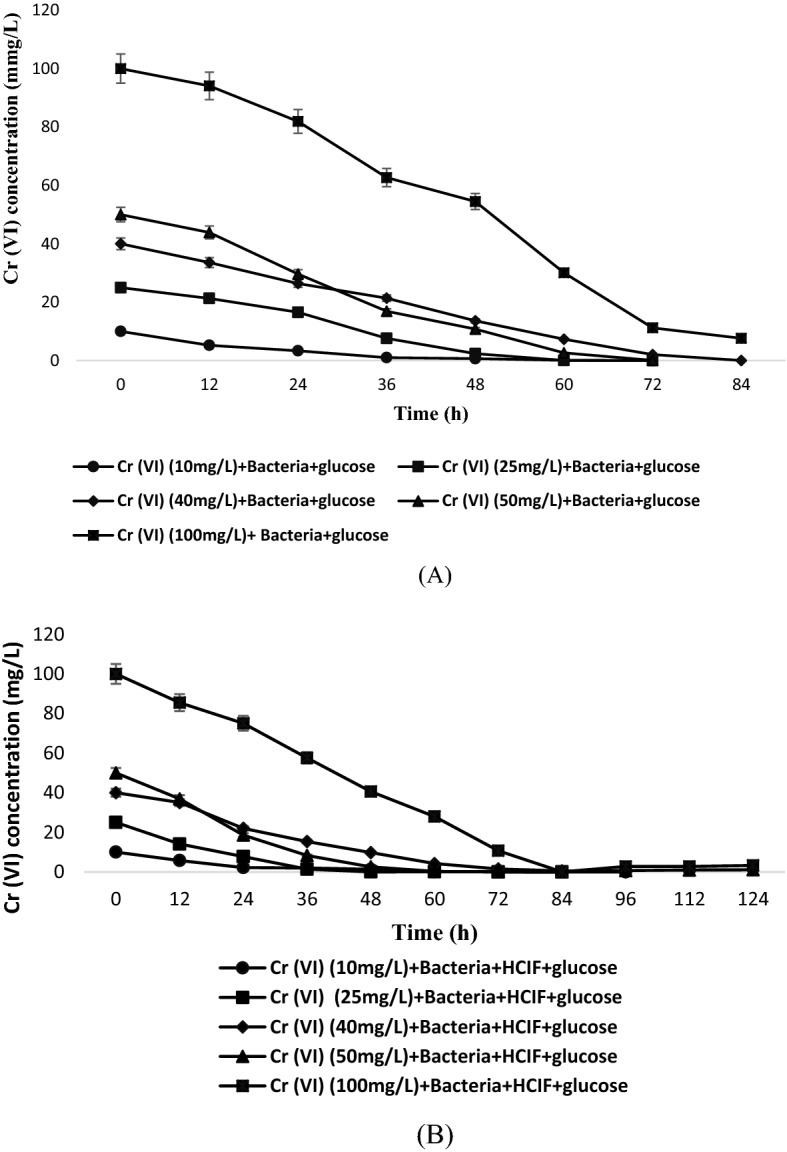
Changes in Cr(VI) concentration (**A**) with glucose as the only substrate (**B**) with glucose and HCIF as the cometabolic substrate.

Experiments were conducted for evaluating the reduction in Cr(VI) concentration with respect to time for the same experimental set as discussed above (Set 1 and Set 2). For Set 1, the results indicated that complete reduction of 10 mg/L and 25 mg/L Cr(VI) concentration was achieved at 40–50 h and 75–76 h, respectively, and similarly for 40 mg/L and 50 mg/L Cr(VI) concentration, the complete reduction was achieved at 80 h. But in case of 100 mg/L Cr(VI) concentration, complete reduction was not achieved till 90 h of experiment (Fig. 2A). At lower concentration of Cr(VI) (10 and 25 mg/L) the maximum reduction was achieved in less time. The reason may be due to the presence of high concentration gradient of Cr in the solution, makes it more difficult to reduce completely^27^. Although glucose was added as a co-metabolic substrate for enhancing the activity of microbes, complete reduction efficiency was not achieved.

The results from the second experimental set (Set 2) showed that for 10 and 25 mg/L Cr(VI) concentration, the reduction was initiated after 15 h and complete reduction was achieved between 30 and 40 h. In case of 40 and 50 mg/L Cr(VI) concentration, the complete reduction was achieved at 60 h. For 100 mg/L Cr(VI) concentration, the reduction was initially slow but after 70 h the reduction was faster and it was completely achieved at 120 h (Fig. 2B). This result was better in comparison to the first experimental set. The results clearly indicated that HCIF was also acting as a co-metabolic substrate in enhancing the reduction efficiency of Cr(VI). The possible mechanism behind improved Cr(VI) reduction is due to coexistence of Serratia sp. with HCIF which helped in reduction of Cr(VI) as well as helped in maintaining the longevity of HCIF. Secondly the strain reduces Fe(III) to Fe(II), resulting in higher dissolved Fe(II) and thus maintaining the structured morphology of HCIF by removing the passivated ferric precipitates on iron surface. It has been confirmed by^28^ also that in the presence of NZVI, the Cr(VI) reduction efficiency by strain DIRB HS01could be improved. Similar type of cometabolic study has been performed by^29^ in which cometabolic degradation of blended biodiesel by a fungal strain Monilliella wahieum Y12^T^ was carried out. The results showed that degradation of petroleum diesel (ULSD) was enhanced when the fungal strain was used with biodiesel. Another experiment conducted by^30^ demonstrated the effect of hydroxypropyl-β-cyclodextrin on cometabolism of phenol and phenanthrene by *Chryseobacterium* sp. The results stated that cometabolic activity of phenol and hydroxypropyl-β-cyclodextrin accelerated the degradation of phenol and had great phenanthrene removal rate. Addition of hydroxypropyl-β-cyclodextrin led to increased solubility and phenanthrene toxicity was also reduced thus had improved cometabolic degradation.

**Figure 3:**
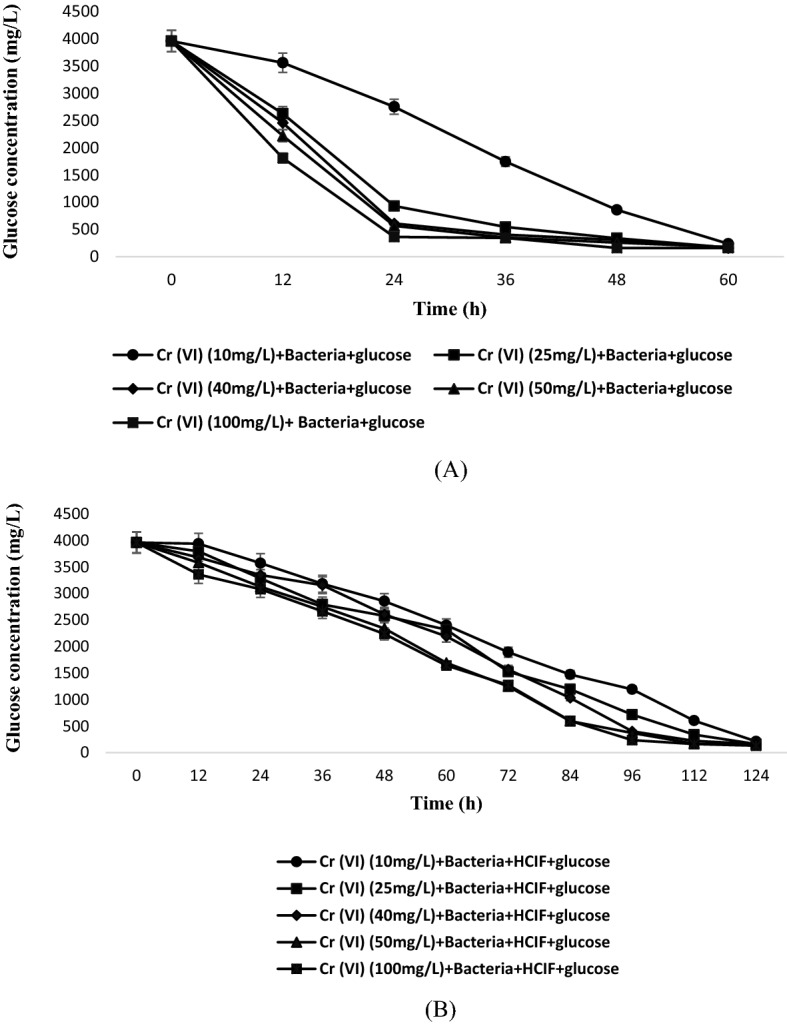
Comparison of glucose concentration with respect to time (**A**) in single substrate system (**B**) in multiple substrate system.

Glucose concentration was also determined at different Cr(VI) concentration at different time interval. Glucose is highly consumed by microbes for their growth and in enhancement of any kind of bacterial activity. Bacterial growth and glucose consumption are inversely proportional to each other. As bacterial growth tends to increase, the glucose consumption also increases because glucose is needed for growth purpose. The similar trend was observed in this study. For Set 1, with doubling of cell number, the glucose consumption increased and was highest at 24 h when the bacterial growth attended its log phase, as discussed above. The glucose concentration started declining after 24 h and it was negligible at 60 h (Fig. 3A). With decrease in glucose concentration, the bacterial growth also declined. The limited amount of glucose available for consumption will automatically destroy the population due to competition for limited amount of food source.

In Set 2, the results indicated that glucose concentration declined slowly with respect to time and the complete decline was observed at 120 h (Fig. 3B).This may be due to the presence of HCIF which also served as a source of growth for microbes, so the glucose consumption in this case was less as compared to set 1 where only glucose was present. As there is presence of two co-metabolic substrate, the burden on substrates may get reduced^19^. The rate kinetics model followed pseudo first order in the case when only glucose was present as substrate (Set 1) and in Set 2 when glucose and HCIF was present as substrates, it followed pseudo second order (Table 1). The rate constant was higher in case of Set 2, when both the substrates were present due to which the Cr(VI) reduced completely. The rate decreased with increase in Cr(VI) concentration in both the Sets 1 and 2. One way ANOVA followed by Duncan’s post hoc test results confirmed that the results were statistically different (Table 2).

**Table 1:** Kinetic analysis for different substrate systems.

Experimental combination	Kinetics			
	1st order rate constant (h^−1^)		2nd order rate constant (mg^−1^ L h^−1^)	
	K	R^2^	K	R^2^
Cr(VI) (10 mg/L) + glucose + bacteria	0.057	0.97	0.0306	0.8789
Cr(VI) (25 mg/L) + glucose + bacteria	0.0388	0.82	0.0071	0.68
Cr(VI) (40 mg/L) + glucose + bacteria	0.0238	0.91	0.0017	0.78
Cr(VI) (50 mg/L) + glucose + bacteria	0.024	0.943	0.0015	0.88
Cr(VI) (100 mg/L) + glucose + bacteria	0.0119	0.938	0.0002	0.88
Cr(VI) (10 mg/L) + glucose + bacteria + HCIF	0.0139	0.9306	0.046	0.954
Cr(VI) (25 mg/L) + glucose + bacteria + HCIF	0.0037	0.729	0.126	0.969
Cr(VI) (40 mg/L) + glucose + bacteria + HCIF	0.0016	0.892	0.0426	0.909
Cr(VI) (50 mg/L) + glucose + bacteria + HCIF	0.0029	0.868	0.0695	0.928
Cr(VI) (100 mg/L) + glucose + bacteria + HCIF	0.0004	0.852	0.0236	0.9118

**Table 2:** One way ANOVA analysis.

Biomass concentration	12 h	24 h	36 h	48 h	60 h
Biotic control	0.144 ± 0.125a	0.691 ± 0.1634a	1.417 ± 0.374a	2.203 ± 0.061a	2.86233 ± 0.080a
10 mg/L	0.139 ± 0.014a	0.204 ± 0.003bc	0.309333 ± 0.028919b	0.612333 ± 0.035796b	0.4573 ± 0.057002b
25 mg/L	0.154667 ± 0.066154a	0.232444 ± 0.021461bc	0.095822 ± 0.026619c	0.042578 ± 0.01024d	0.029522 ± 0.005303c
40 mg/L	0.056667 ± 0.015275b	0.266 ± 0.012b	0.131 ± 0.009849c	0.145333 ± 0.049075c	0.02287 ± 0.001499c
50 mg/L	0.030489 ± 0.004575b	0.059444 ± 0.017519c	0.038778 ± 0.014206c	0.049667 ± 0.027099d	0.026333 ± 0.005774c
100 mg/L	0.019667 ± 0.002309b	0.258778 ± 0.213544b	0.106611 ± 0.131977c	0.076889 ± 0.019357d	0.017444 ± 0.001711c

All values are represented as mean ± SD. Different alphabets represents difference in mean according to one way ANOVA followed by Duncan’s post hoc test at probability level (p ≤ 0.05).

### Kinetics of microbial growth in single and tertiary substrate system

**Figure 4:**
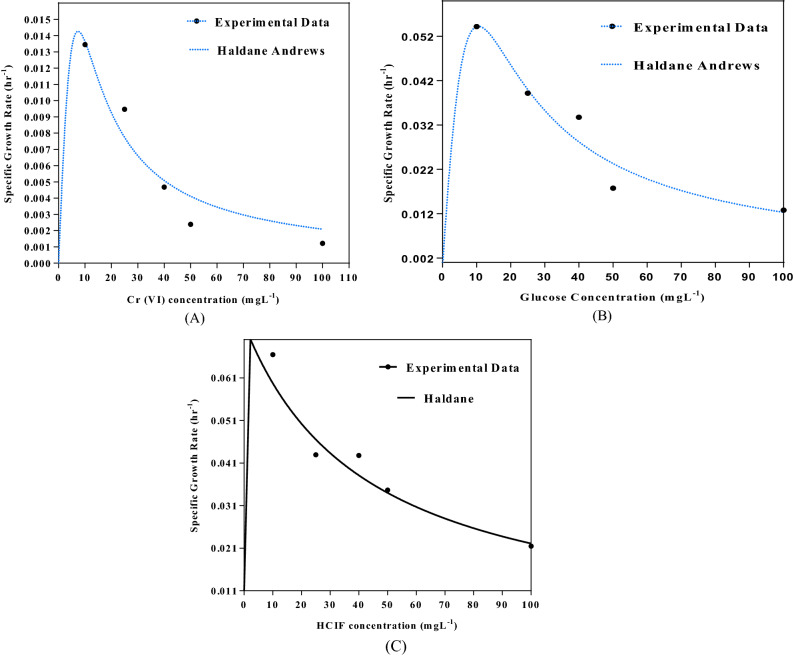
Growth Kinetics modeling in single substrate system (**A**) with respect to Cr(VI) concentration, (**B**) glucose concentration, (**C**) HCIF concentration.

The experimentally acquired data for the specific growth rate of the strain at its growth in single substrate system consisting of different chromium concentration as substrate 1, different glucose concentration as substrate 2 and different HCIF concentration as substrate 3 were used to fit the stated kinetic models using the nonlinear regression analysis in GraphPad Prism 6 software for evaluating the kinetic parameters. Among other inhibitory growth kinetic models, Haldane Andrews’s model gave the best fit for the experimentally acquired data for all substrates (Fig. 4A–C). The R^2^ value for only chromium as a substrate was found to be 0.977, µmax value 0.002346 and the inhibition coefficient value to be 134.12. When only glucose was added as a substrate, the µmax and inhibition coefficient were found to be 0.1129 and 1.01 respectively with R^2^ 0.9916. In case of HCIF, the µmax and the inhibition coefficient were 0.1923 and 11.99 respectively and R^2^ value was 0.9876. The values of specific growth rate obtained from fitting the values of single substrate system was later applied to the various inhibitory growth models to check for the cometabolic activity in tertiary substrate system.

**Figure 5:**
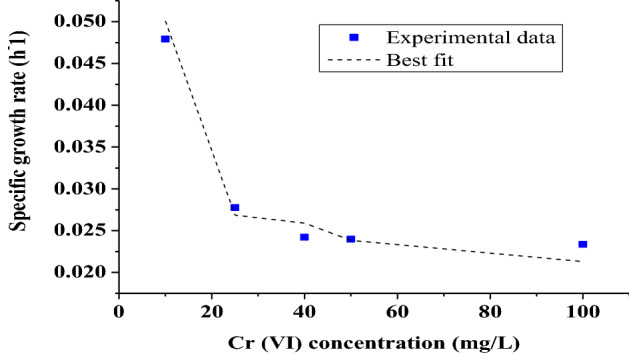
Growth kinetic modelling in tertiary substrate system.

Out of the other inhibitory models used for fitting, Monod model was best fitted for the growth kinetics in tertiary substrate system in order to evaluate the interaction between the three co-metabolic substrate (i.e. HCIF and glucose and chromium) in enhancing Cr(VI) reduction. Previous studies^31^ showed that addition of some co-metabolic substrate to the system was effective in dealing the degradation of refractory compounds. To understand the co-metabolism between glucose, Cr(VI) and HCIF in the tertiary system, Eq. (3) was used for the cell growth kinetic analysis. The relative kinetic parameters obtained from the analysis are presented in Table 3.The model was best fitted with the R^2^ value of 0.988 (Fig. 5).

**Table 3:** Growth kinetic parameters of *Serrtia* sp. for tertiary substrate system.

Kinetic constants	Different Cr(VI) concentration				
	10 mg/L	25 mg/L	40 mg/L	50 mg/L	100 mg/L
${K}_{2 C }{S}_{P}$	0.287	0.655	0.834	0.761	0.934
${K}_{3 C}{S}_{h}$	0.015	0.047	0.062	0.059	0.114
${K}_{4C}{S}_{P}{S}_{h}$	0.0249	0.068	0.083	0.061	0.154
${K}_{2P}{S}_{C}$	0.586	1.221	1.146	1.452	1.625
${K}_{3P}{S}_{h}$	0.489	0.812	0.741	1.332	1.524
${K}_{4P}{S}_{C}{S}_{h}$	0.04	0.042	0.092	0.289	0.451
${K}_{2h}{S}_{C}$	0.017	0.052	0.035	0.124	0.1
${K}_{3h}{S}_{P}$	0.08	0.094	0.103	0.121	0.267
${K}_{4h}{S}_{C}{S}_{P}$	0.041	0.057	0.1149	0.201	0.415

The values of K_2_CS_P_ (Inhibition of glucose in the presence of Cr(VI)), K_3_C S_h_ (Inhibition of glucose in the presence of HCIF) and K_4_C S_P_ S_h_ (Inhibition of glucose in the presence of Cr(VI) and HCIF) increased with increasing Cr(VI) concentration. Amongst the three kinetic constants, the value of K_3_C S_h_ is lower as compared to K_4_C S_P_ S_h_ and K_2_C S_P_. Lower values of K_3_C S_h_, indicates that the inhibition of glucose consumption is low in presence of HCIF due to the fact that HCIF was also providing food source for the microbial growth other than glucose. The values of K_2_C S_P_ and K_4_C S_P_ S_h_ revealed that when only Cr was present (K_2_C S_P_) the inhibition on glucose consumption was higher as compared to when both Cr and HCIF (K_4_C S_P_ S_h_) were present. Similarly, amongst the other three kinetic constant values K_2_P S_C_ (Inhibition of Cr(VI) reduction in the presence of glucose), K3P Sh (Inhibition of Cr(VI) reduction in the presence of HCIF) and K_4_P S_P_S_C_ (Inhibition of Cr(VI) reduction in the presence of glucose and HCIF), K_2_P S_C_ values were higher which indicates that when only glucose was present (K_2_P S_C_) inhibition on Cr reduction was higher followed by inhibition on Cr reduction when only HCIF was present (K_3_P S_h_). But when both substrates (glucose and HCIF) were present (K_4_P S_P_S_C_), the inhibition on Cr reduction was lowest. Inhibition of HCIF consumption due to glucose (K_2_h S_C_) is lowest as compared to inhibition of HCIF consumption in presence of both Cr and glucose (K_4_h S_P_S_C_) and when only Cr was present (K_3_h S_P_). When HCIF and glucose were present, microbes were utilizing both glucose and HCIF for their growth. The higher values of K_2_P and K_3_P suggested high degree of inhibition of glucose and HCIF on Cr(VI) reduction and diauxic growth of the strain. Similarly lower values of K_2_C and K_3_C indicated the negligible impact of Cr(VI) imposed to glucose and HCIF consumption. It has been studied by^32^ that for the growth of cell on mixed substrates, there are categorization of the interaction between the growth substrates which is defined as competitive, noncompetitive and uncompetitive inhibition of growth. On the basis of these observations, the results showed that glucose and Cr(VI) were not structurally similar, so uncompetitive inhibition was the best interpretation to the interaction between glucose and Cr(VI). The interaction constants K_2_h and K_3_h (inhibition of iron due to glucose and Cr(VI) respectively) were one to threefold larger than the corresponding K_2_C and K_3_C. Thus it can be inferred that another possible mixed pattern of interaction exists involving both competitive and uncompetitive inhibition. The values of kinetic constants K_4_C, K_4_P and K_4_h indicated that K_4_P and K_4_h values are lower as compared to the value of K_4_C. This may be due to the reason that the inhibition factor was more in case when there were presence of Cr and HCIF which poses competitive inhibition. The results confirmed that both glucose and HCIF as a co-metabolic substrate had a great role in Cr(VI) reduction, but when HCIF was added as another substrate, it increased the reduction efficiency of the system. The strain utilized glucose as well as HCIF for maintaining their activity and for their growth. It has been reported by^33,34^ that two substrates (glucose and biphenyl) had great role in bioremediation of PBDEs. However, a large number of reports^35^ indicated that optimum dose of co-substrates is essential to use because excessive dose of any of the co-substrates hampers the degradation efficiency and was attributed for the inhibition between the substrates. Excess dosage of substrates would cause self-inhibition on growth of the cell, thus decrease reduction efficiency. Therefore, it is extremely important to choose appropriate co-substrate and optimum dosage of substrate.

## Conclusion

The efficiency of Cr(VI) reduction by *Serratia* sp. was improved by cometabolism activity. Meanwhile, it is known that presence of diversity of carbon sources impose effects on the interactive activity of metabolic pathways and substrates. Glucose had great role as anti-competitive inhibitor in Cr(VI) reduction. However, on the other side, another substrate HCIF had both competitive and uncompetitive inhibition on Cr(VI) reduction by enhancing the strain ability to help in complete reduction. Both glucose and HCIF as a co-metabolic substrate had a great role in Cr(VI) reduction, but when HCIF was added as another substrate, it increased the reduction efficiency of the system and combination of HCIF and glucose showed least inhibition to hexavalent chromium reduction by *Serratia* sp. The outcomes of the current work enlightens a noteworthy approach to carry out treatment of other compounds as well and a great finding for enhancing long term performance of ZVI PRBs in combination with *Serratia* Sp. for remediation of Cr(VI) contaminated sites.

## References

[1] Sathvikaa T, Balajia S, Chandraa M, Sonia A, Rajesh V, Rajesh N (2019). A co-operative endeavor by nitrifying bacteria *Nitrosomonas* and Zirconium based metal organic framework to remove hexavalent chromium. Chem. Eng. J..

[2] Pradhan D, Sukla LB, Sawyer M, Rahman PKSM (2017). Recent bioreduction of hexavalent chromium in wastewater treatment: A review. J. Ind. Eng. Chem..

[3] Wua M, Lib Y, Lia J, Wanga Y, Xua H, Zhaoa Y (2019). Bioreduction of hexavalent chromium using a novel strain CRB-7 immobilized on multiple materials. J. Hazard. Mater..

[4] Di Palma L, Verdone N, Vilardi G (2018). Kinetic modeling of Cr(VI) reduction by nZVI in soil: The influence of organic matter and manganese oxide. Bull. Environ. Contam. Toxicol..

[5] Brasili E, Bavasso I, Petruccelli V, Vilardi G, Valletta A, Dal Bosco C, Gentili A, Pasqua G, Di Palma L (2020). Remediation of hexavalent chromium contaminated water through zero-valent iron nanoparticles and effects on tomato plant growth performance. Sci. Rep..

[6] Lin CJ, Wang SL, Huang PM, Tzou YM, Liu JC, Chen CC, Chen JH, Lin C (2009). Chromate reduction by zero-valent Al metal as catalyzed by polyoxometalate. Water Res..

[7] Mpouras T, Polydera A, Dermatas D, Verdone N, Vilardi G (2020). Multi wall carbon nanotubes application for treatment of Cr (VI)-contaminated groundwater; Modeling of batch & column experiments. Chemosphere.

[8] Scharf B, Clement CC, Zolla V, Perino G, Yan B, Elci SG, Purdue E, Goldring S, Macaluso F, Cobelli N, Vachet RW, Santambrogio L (2014). Molecular analysis of chromium and cobalt-related toxicity. Sci. Rep..

[9] Desai C, Jain K, Madamwar D (2008). Evaluation of in vitro Cr(VI) reduction potential in cytosolic extracts of three indigenous *Bacillus* sp. isolated from Cr(VI) polluted industrial landfill. Bioresour. Technol..

[10] Srivastava S, Yadav GK, Sinha A, Mishra BK (2014). Comparative study for reduction of hexavalent chromium by high carbon iron filings (HCIF) and electrolytic Iron:mass transfer limitations. Asian. J. Chem..

[11] Banerjeea S, Misraa A, Chaudhury S, Dama B (2019). A *Bacillus* strain TCL isolated from Jharia coalmine with remarkable stress responses, chromium reduction capability and bioremediation potential. J. Hazard. Mater..

[12] Gong K, Hu Q, Yao L, Li M, Sun D, Shao Q, Qiu B, Guo Z (2018). Ultrasonic pretreated sludge derived stable magnetic active carbon for Cr (VI) removal from wastewater. ACS Sustain. Chem. Eng..

[13] Huang J, Li Y, Cao Y, Peng F, Cao Y, Shao Q, Liu H, Guo Z (2018). Hexavalent chromium removal over magnetic carbon nanoadsorbents: Synergistic effect of fluorine and nitrogen co-doping. J. Mater. Chem. A.

[14] Singh R, Kumar M, Bishnoi NR (2016). Development of biomaterial for chromium (VI) detoxification using *Aspergillus flavus* system supported with iron. Ecol. Eng..

[15] Arévalo-Rangel DL, Cárdenas-González JF, Martínez-Juárez VM, Acosta-Rodríguez I (2013). Hexavalent chromate reductase activity in cell free extracts of *Penicillium* sp. Bioinorg. Chem. Appl..

[16] Robins KJ, Hooks DO, Rehm BH, Ackerley DF (2013). *Escherichia coli* NemA is an efficient chromate reductase that can be biologically immobilized to provide a cell free system for remediation of hexavalent chromium. PLoS ONE.

[17] Ge S, Ge S, Zhou M, Dong X (2015). Bioremediation of hexavalent chromate using permeabilized *Brevibacterium* sp. and *Stenotrophomonas* sp. cells. J. Environ. Manag..

[18] Qu M, Chen J, Huang Q, Chen J, Xu Y, Luo J, Wang K, Gao W, Zheng Y (2018). Bioremediation of hexavalent chromium contaminated soil by a bioleaching system with weak magnetic fields. Int. Biodeterior. Biodegrad..

[19] Lv YC, Li LH, Chen YC, Tang ZH, Hu YY (2016). Effects of glucose and biphenyl on aerobic cometabolism of polybrominated diphenylethers by *Pseudomonas putida*: Kinetics and degradation mechanism. Int. Biodeterior. Biodegrad..

[20] Zhoua J, Lib H, Chenc X, Wand D, Maia W, Suna C (2017). Cometabolic degradation of low-strength coking wastewater and the bacterial community revealed by high-throughput sequencing. Bioresour. Technol..

[21] Jamshidian H, Khatami S, Mogharei A, Vahabzadeha F, Nickzad A (2013). Cometabolic degradation of para-nitrophenol and phenol by *Ralstonia eutropha* in a Kissiris immobilized cell bioreactor. Korean J. Chem. Eng..

[22] Shi S, Qu Y, Zhou H, Ma Q, Ma F (2015). Characterization of a novel cometabolic degradation carbazole pathway by a phenol-cultivated *Arthrobacter *sp. W1. Bioresour. Technol..

[23] Heidari H, Sedighi M, Zamir SM, Shojaosadati SA (2017). Bisphenol A degradation by *Ralstonia eutropha* in the absence and presence of phenol. Int. Biodeterior. Biodegrad..

[24] Wang W, Wu B, Pan S, Yang K, Hu Z, Yuan S (2017). Performance robustness of the UASB reactors treating saline phenolic wastewater and analysis of microbial community structure. J. Hazard. Mater..

[25] Upadhyay S, Tarafdar A, Sinha A (2020). Assessment of *Serratia* sp. isolated from iron ore mine in hexavalent chromium reduction: kinetics, fate and variation in cellular morphology. Environ. Technol..

[26] Jiang C, Xu X, Megharaj M, Naidu R, Chen Z (2015). Inhibition or promotion of biodegradation of nitrate by *Paracoccus* sp. in the presence of nanoscale zero-valent iron. Sci. Total Environ..

[27] Das, A. P. & Mishra, S. Biodegradation of the metallic carcinogen hexavalent chromium Cr (VI) by an indigenously isolated bacterial strain. *J. Carcinog.* **9**(6). 10.4103/1477-3163.63584 (2010). 10.4103/1477-3163.63584 PMC2957624 20976016

[28] Shi Z, Shen W, Yang K, Zheng N, Jiang X, Liu L, Yang D, Zhang L, Ai Z, Xie B (2019). Hexavalent chromium removal by a new composite system of dissimilatory iron reduction bacteria *Aeromonas hydrophila* and nanoscale zero-valent iron. Chem. Eng. J..

[29] Ye C, Ching TH, Yoza BA, Masutani S, Li QX (2017). Cometabolic degradation of blended biodiesel by *Moniliella wahieum* Y12T and *Byssochlamys nivea* M1. Int. Biodeterior. Biodegrad..

[30] Xiao M, Xiangyang Y, Hengjun G, Honglei M, Yanfeng Q, Kun L, Xia H, Sun M (2019). Effect of hydroxypropyl-β-cyclodextrin on the cometabolism of phenol and phenanthrene by a novel *Chryseobacterium* sp. Bioresour. Technol..

[31] Field JA, Sierra-Alvarez R (2008). Microbial transformation and degradation of polychlorinated biphenyls. Environ. Pollut..

[32] Kargi F, Shuler ML (1992). Bioprocess Engineering: Basic Concepts.

[33] Lu M, Zhang ZZ, Wu XJ, Xu YX, Su XL, Zhang M, Wang JX (2013). Biodegradation of decabromodiphenyl ether (BDE-209) by a metal resistant strain, *Bacillus cereus* JP12. Bioresour. Technol..

[34] Shi G, Yin H, Ye J, Peng H, Li J, Luo C (2013). Aerobic biotransformation of decabromodiphenyl ether (PBDE-209) by *Pseudomonas aeruginosa*. Chemosphere.

[35] Xin J, Liu X, Liu W, Zheng XL (2014). Aerobic transformation of BDE-47 by a *Pseudomonas putida* sp. strain TZ-1 isolated from PBDEs-contaminated sediment. Bull. Environ. Contam. Toxicol..

